# Supplementary material for: A 5Ad Dietary Protocol for Functional Bowel Disorders
Source: Nutrients. 2019 Aug 17;11(8):1938. doi: 10.3390/nu11081938 (PMC6722668; doi:10.3390/nu11081938)
Supplement: Supplementary file 1 [file nutrients-11-01938-s001.zip › Supplementary Material 1_5Ad Dietary Protocol.pdf]

5Ad Dietary Protocol for Functional Bowel Disorders

**Group 1**	Chicken, turkey, oily fish, white fish, seafood, eggs *(boiled or poached),* mature/hard cheese *(e.g. cheddar or gouda)*, low-fat/fat-free natural yoghurt *(just plain)*
**Group 2**	Potato, sweet potato *(for other tubers please consult with the researchers)*
**Group 3**	Walnut, cashew, almond, pecan, macadamia, hazelnut, brazil nut, pine nut, sunflower seeds, pumpkin seeds, sesame seeds *(all unroasted and unsalted)*
**Group 4**	Rocket salad, lettuce, kale, spinach, tomato, cucumber, carrot, aubergine, courgette, olives
**Group 5**	Strawberries, blueberries, raspberries, blackberries, kiwi fruit, banana, orange, tangerine, plum, apricot, grapes, melon *(excluding watermelon)*, prickly pear, fig, dates *(all can be fresh, frozen, or dried, but unsweetened and without any additives whatsoever)*

**Flavourings***	Honey, maple syrup, olive oil *(cold pressed, extra virgin)*, rapeseed oil *(cold pressed, extra virgin)*, salt

*****Please note that the above flavourings are allowed on the protocol but are not classed as one of the “5Ad” groups

Disclaimer: Please not that this protocol is not a cure for FBDs, but is designed to help you find the optimal diet for symptom relief, therefore improving your quality of life. If the protocol is to be followed beyond the intervention period, please consult with the researchers beforehand.

Instructions

1) **Please consume at least 1 item from each of the 5 groups every day**
2) You can have a mixture of the items in each group as well
3) Please use only **boiling**, **steaming, baking, grilling**, **microwaving**, or **pressure-cooking** methods to prepare your food
4) Please be strict and be sure to consume only the selected foods within the table
5) Consume foods in their natural form (e.g. crisps do not count as potatoes)
6) Fresh fruit can be eaten in unlimited amounts, but consume dried fruit (e.g. dates) in moderation
7) Please try to limit mature cheese to <75g per day
8) If consumed, please limit honey or maple syrup to 1-2 tsp per day and limit olive oil or rapeseed oil to 1 tbsp per day
9) Please add 1 tsp salt per day to your meals if you do not consume any pickled foods (e.g. olives) or mature cheese that day
10) Coffee (both caffeinated and decaffeinated) is not permitted. Tea (black or white) is allowed within a maximum of 5 cups per day
11) Cereal-based alcohol should be avoided, and distilled or grape-based alcohol can be consumed in moderation

Tips and Recommendations

1) There are no set recipes for the protocol, and you can make your own recipes, but for meal ideas please visit: https://drive.google.com/drive/folders/1kzEgVhGIf0jekIBjrcnM18bx-ahU-VLJ?usp=sharing
2) Try to consume around 1kg fresh fruit and/or vegetables per day (**from the permitted list**)
3) Aim to drink 6-8 glasses of water per day
4) Take care when eating out to stick to the protocol – a jacket potato with cheddar cheese and salad, or grilled chicken/fish with salad and new potatoes are good options and try to order a side salad instead of chips, for example. Plain nuts and fresh fruit are great snacks from the supermarket if you need to eat on the go
5) Try to prepare food in advance for eating at work to avoid deviating from the protocol
6) To obtain higher nutritional value from small seeds (e.g. sesame), it is best to crush them with a home mixer or buy them ready crushed
7) Moderate physical activity is recommended

Please do not hesitate to contact the researchers if you have any questions.

Philippa Stribling: p.stribling@uos.ac.uk **+447903 862 556**
Dr Fandi Ibrahim: f.ibrahim@uos.ac.uk **+447799 821 928**

Disclaimer: Please not that this protocol is not a cure for FBDs, but is designed to help you find the optimal diet for symptom relief, therefore improving your quality of life. If the protocol is to be followed beyond the intervention period, please consult with the researchers beforehand.

Post-intervention instructions

The data of each participant is to be assessed by the two researchers based on the change in abdominal symptoms scores and the participant's feedback. Upon this assessment, either of the two following scenarios will apply:

1) If the participant does not see any significant improvement, they are instructed to go back to their usual diet/treatment or consult with their GP/Dietitian/Nutritionist.

2) If the participant sees a significant improvement and they are happy to continue on the diet, the following instructions are handed to them.

Post-improvement instructions for the 5Ad Dietary Protocol

A. You can continue to follow the protocol for as long as you want, but make sure to:
   - ✓ Consume at least one item daily from each of the five groups in a similar manner to the intervention week.
   - ✓ Vary between the items within each group over a week period on average (for example, from group one, you may eat chicken, the other day eat turkey, another day eat fish, and so on) - see NHS advice on large fish consumption.
   - ✓ For nuts and seeds, having a mixture of ≥ 100 g/day is a good thing to aim for.
   - ✓ For the fruit and vegetable groups, we also recommend a daily mixture or variation over a week, based on your preferences, the seasonality, and cost.
   - ✓ If you do not include oily fish products, it is best to have vitamin D3 supplement, particularly during winter months (10 ug/day).
   - ✓ If you do include oily fish products, then you may reduce the vitamin D3 supplement dose to a 5ug/day.
   - ✓ If you cannot tolerate any low-lactose or lactose-free dairy products, calcium supplements may be used upon consultation with your health practitioner.

B. <u>In principle, you can start to introduce your usual other foods as you wish but one by one; those that you can tolerate can remain in your diet, and those you cannot tolerate can be excluded. We recommend that you follow this guidance:</u>

- ✓ You must not introduce any foods that you have been known to be allergic too or that were originally excluded for medical reasons (e.g. gluten for coeliac disease).
- ✓ Make boiling and steaming the most common ways of cooking.
- ✓ You can introduce herbs and spices back into your diet. Most spices and herbs should be tolerated, with the exception of garlic and onion, which should be introduced carefully and in small amounts to see how they affect you.
- ✓ You can introduce other fruits and vegetables one at a time and see how you feel with them.
- ✓ You can start to introduce lean cuts of beef, lamb, and/or pork in limited amounts (see NHS advice on red meat consumption).
- ✓ Bird meat, such as duck, goose, or any game meat can be enjoyed as well.
- ✓ You can introduce pseudo-cereals, such as buckwheat as these are old crops and likely to be tolerated, but we have no evidence for that yet.
- ✓ Leave the introduction of all <u>cereals (including oats, wheat, maize, rice, etc), legumes (including soy)</u> and <u>cabbage</u> (and its plant relatives, e.g. <u>Brussels sprouts, cauliflower</u>) as late as you can, and bear in mind that you may never be able to eat some of these again regularly without developing symptoms – we are not all genetically adapted to the modern diet.
- ✓ If you introduced cereals in their common way (processed), and find out that you are intolerant to them, you may buy them as whole seeds, soak them over night, and boil them for breakfast (wheat, barely, sorghum, oats, brown rice); not as ready porridge, flakes or shredded wheat, for example.
- ✓ It is unlikely that you will tolerate legumes/pulses (including soy), but if you would like to introduce any, use small amounts from the same type or the same mix regularly (less than 100 g cooked legumes/pulses per day or every other day). If you

stop eating them for a few days, and then decide to eat them again, you may experience the same symptoms.

- ✓ The same advice above applies to the cruciferous family (cabbage, etc.) but kale should be fine.
- ✓ Flaxseed and chia seeds can be introduced, but carefully, to assess your tolerance to them.
- ✓ To obtain higher nutritional value from small seeds (e.g. sesame, chia, or flaxseed), it is best to crush them with a kitchen mixer or to buy them ready crushed (make sure to read the ingredient list).
- ✓ 100% nut/seed butters (from nuts/seeds included within the 5Ad Dietary Protocol) should be fine to consume.
- ✓ You should continue to avoid coffee of all kinds, but you can drink tea regularly, with or without milk.
