# Supplementary material for: A 5Ad Dietary Protocol for Functional Bowel Disorders
Source: Nutrients. 2019 Aug 17;11(8):1938. doi: 10.3390/nu11081938 (PMC6722668; doi:10.3390/nu11081938)
Supplement: Supplementary file 1 [file nutrients-11-01938-s001.zip › Supplementary Material 2_Nutritional Analysis.pdf]

Dietary Analysis

Average of 7 day log

27 year old female, 70kg, 1.68m | **P_F 5Ad_Plan**

2nd Nov 2018 - 8th Nov 2018

NUTRIENT	AVG. INTAKE	TARGET	LIMITS	LOWER LIMIT / ACTUAL INTAKE / YOUR TARGET / UPPER LIMIT
**- ENERGY -**				
**Energy(Kcal)**	**1876kcal** (85%)	2206kcal		
**- MACRONUTRIENTS -**				
**Carbohydrate**	**207g** (75%)	276g		
**Protein**	**76g** (144%)	53g		
**Fat**	**84g**		<86g	
**Water**	**1356g**			N/A
› ***water from drinks***	**0g**			N/A
**Alcohol**	**0g**		<15.8g	
**- CARBOHYDRATE -**				
**Starch**	**98g**			N/A
**Oligosaccharide**	**0.27g**			N/A
**Fibre**	**36.7g** (122%)	30g		
**NSP**	**25.9g**			N/A
**Sugars**	**108g**			N/A
**Free Sugars**	**0g**		<27.6g	
**- LIPID COMPONENTS -**				
**Saturated Fat**	**16.9g**		<27g	
› ***monounsaturated fat***	**34.2g** (107%)	31.9g		
**Polyunsaturated fat**	**24g**		>6.5g <24.5g	
› ***omega3(n-3)***	**3.2g**		>0.49g	
› ***omega6(n-6)***	**20.8g**		>2.5g	
**Trans-fatty acids**	**0.38g**		<4.9g	
**Cholesterol**	**177mg**			N/A
**- MINERALS & TRACE ELEMENTS -**				
**Sodium**	**1320mg** (82%)	1600mg	>575mg <2400mg	
**Potassium**	**5753mg** (164%)	3500mg	>2000mg	
**Chloride**	**2773mg** (110%)	2500mg		
**Calcium**	**730mg** (104%)	700mg	>400mg	
**Phosphorus**	**1425mg** (259%)	550mg		
**Magnesium**	**534mg** (197%)	270mg	>150mg	
**Iron**	**13.7mg** (92%)	14.8mg	>8mg	

Zinc	**9.8mg** (139%)	7mg	>4mg	
Copper	**2.6mg** (215%)	1.2mg		
Manganese	**5mg**		>1.4mg	
Selenium	**50ug** (82%)	60ug	>40ug	
Iodine	**136ug** (96%)	140ug	>70ug	
- VITAMINS -				
Vitamin A (ret eq)	**4258ug** (709%)	600ug	>250ug	
Vitamin D	**4.1ug** (41%)	10ug		
Vitamin E	**33.5mg**		>3mg	
Vitamin K_1	**236ug** (336%)	70ug		
Thiamin (B_1)	**1.5mg** (192%)	0.8mg	>0.23mg	
Riboflavin (B_2)	**1.1mg** (99%)	1.1mg	>0.8mg	
Niacin total (B_3)	**30.9mg** (212%)	14.6mg	>9.7mg	
Pantothenic Acid (B_5)	**6.1mg** (122%)	3-7mg		
Vitamin B_6	**1.9mg** (165%)	1.1mg	>0.84mg	
Folates (B_9) Total	**382ug** (191%)	200ug	>100ug	
Vitamin B_{12}	**4.2ug** (281%)	1.5ug	>1ug	
Biotin (B_7)	**49ug** (46%)	10-200ug		
Vitamin C	**226mg** (566%)	40mg	>10mg	
- OTHER -				
GL	**106**			N/A
Caffeine	**0mg**			N/A

Figures from UK: SACN 2017 / COMA guidelines for female, 25-35 years old

Generated by Nutritics v5.096 on 10th Aug 2019

Macronutrient Analysis

	CARBOHYDRATE	SUGARS	PROTEIN	FAT	SATFAT	ALCOHOL
Intake	206.9g	108.4g	76g	84.2g	16.9g	0g
g/kg body-weight	3	1.5	1.1	1.2	0.2	0
Kilocal	814	434	304	758	153	0
Kilocal %	43.4%	23.1%	16.2%	40.4%	8.1%	0%

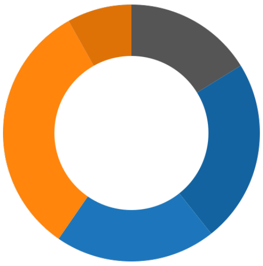

43.4% Carbs
16.2% Protein
40.4% Fat

DAYS 1

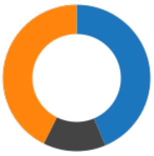

43.6%
13.7%
42.6%
0%

DAYS 2

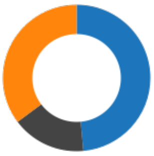

48.5%
16.2%
35.2%
0%

DAYS 3

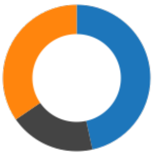

46.6%
18.9%
34.6%
0%

DAYS 4

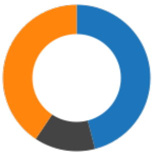

46%
13.4%
40.6%
0%

DAYS 5

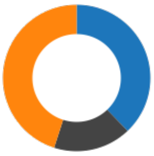

37.9%
17.1%
45.1%
0%

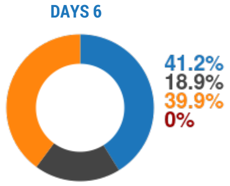

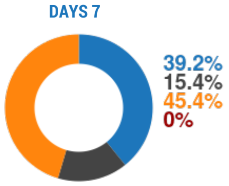

Notes

This report represents the dietary analysis of the average weekly intake of a female who followed the 5Ad Dietary Protocol strictly, with 1.78 m height, and a target weight of 70 kg. The middle vertical reference line represents the DRVs/RNIs as set by the SACN committee, UK (Figures from UK: SACN 2017 / COMA guidelines for female, 25-35 years old).

- All nutrients were compared against their respective DRVs/RNIs and nothing was red-flagged in terms of the sufficiency nor excessive intake.

- The percentages of the macronutrient distribution of the 5Ad Diet is as close as possible to SACN guidelines, (43-16-40) vs (50-15-35) of total energy intake for CHO-Protein-Fat, respectively.

- Fibre is even slightly higher than the recommended but it is still a safe level and all from natural sources (nuts, seeds, fruits, vegetables including tubers).

- There is zero added sugar (zero free sugars, UK terminology).

- The total fat content, even if it is slightly higher, is from all natural sources and poly- and monounsaturated fat are at an optimal level; saturated fat is low and omega 3 and 6 fatty acids meet the requirements.

- The protein intake is within the guidelines and when checked per kg body weight, it is still a safe level.

- All micronutrient requirements are met.

- Vitamin D is a special case and attention has been paid to this in the post-intervention guidance (which is part of the protocol in the long-term), as well as calcium intake.

- Na/K ratio is about ideal; although we recognise that this ratio is still a debatable matter.
