# Supplementary figures and images for: A 5Ad Dietary Protocol for Functional Bowel Disorders
Source: Nutrients. 2019 Aug 17;11(8):1938. doi: 10.3390/nu11081938 (PMC6722668; doi:10.3390/nu11081938)

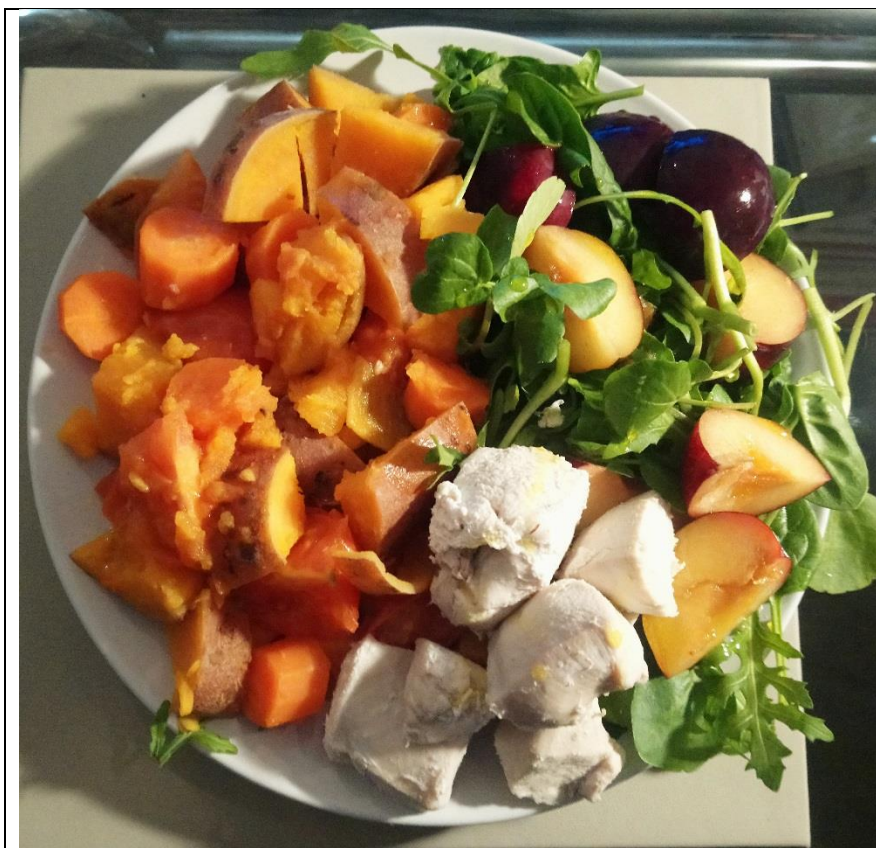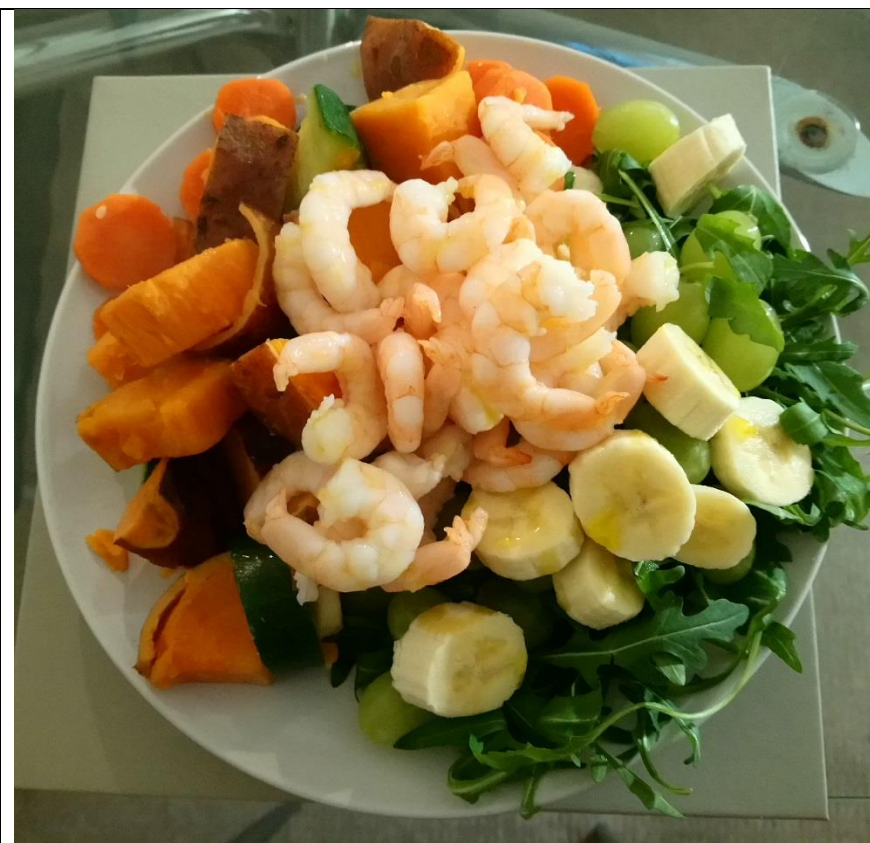

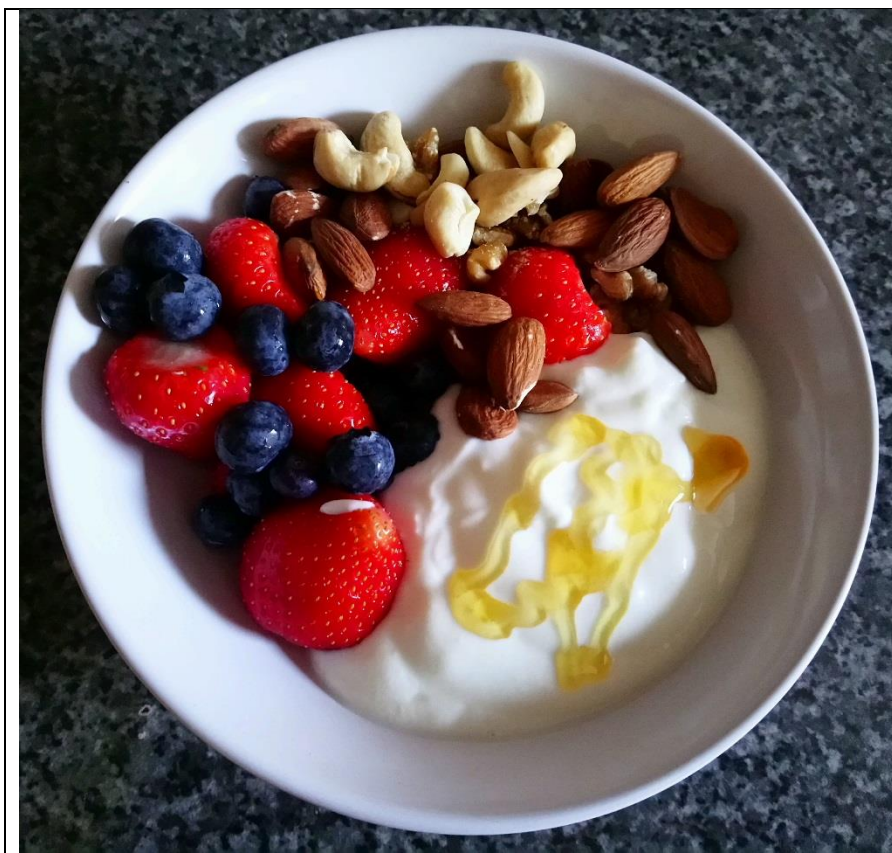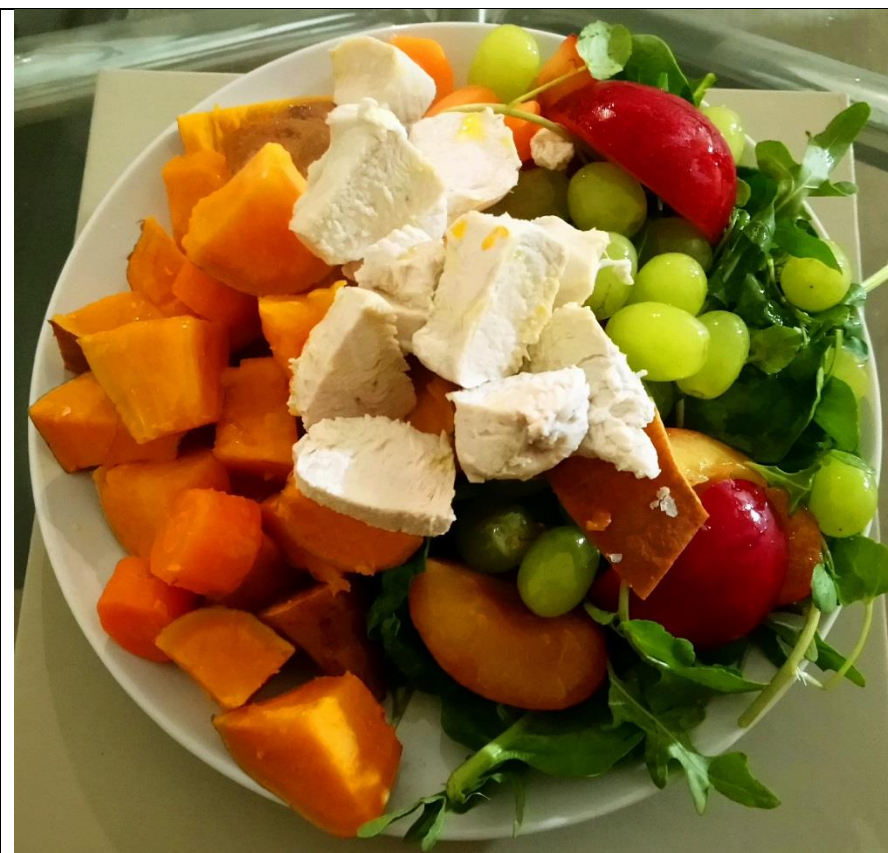

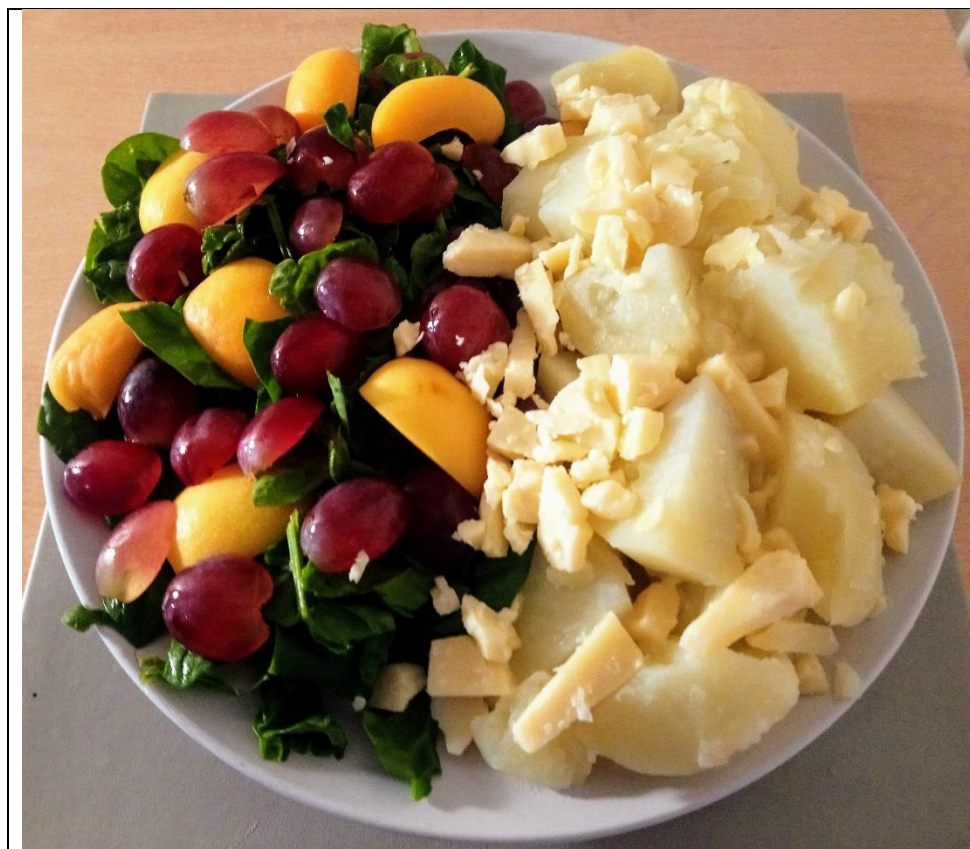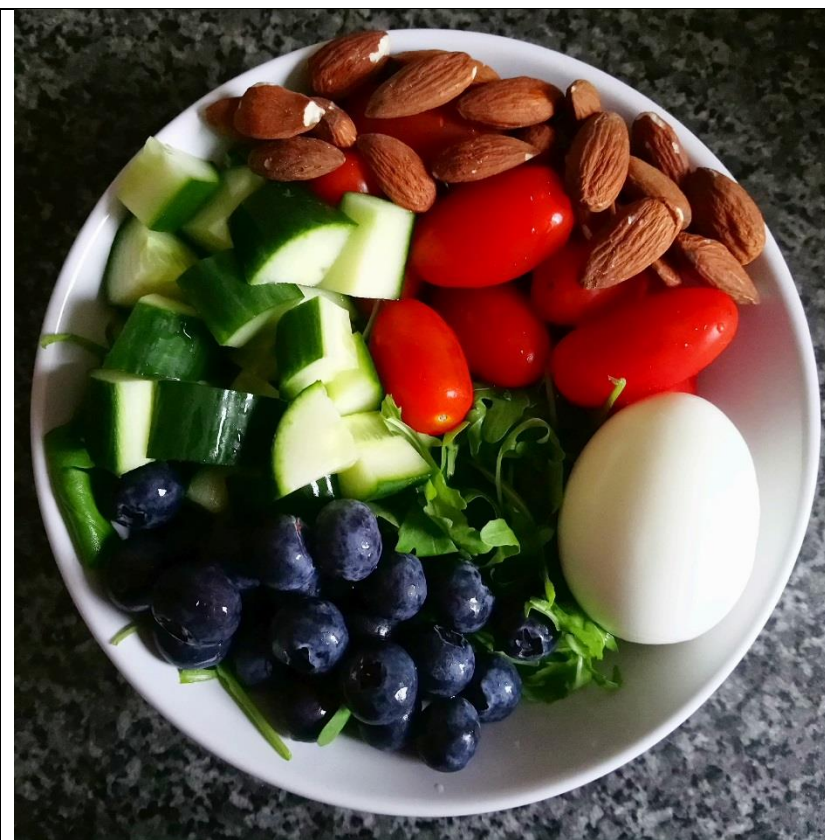

Supplement: Supplementary file 1 [file nutrients-11-01938-s001.zip › Supplementary Material 3_Meal examples.pdf]
